# Correlation between differential drought tolerability of two contrasting drought-responsive chickpea cultivars and differential expression of a subset of *CaNAC* genes under normal and dehydration conditions

**DOI:** 10.3389/fpls.2015.00449

**Published:** 2015-06-19

**Authors:** Kien Huu Nguyen, Chien Van Ha, Yasuko Watanabe, Uyen Thi Tran, Maryam Nasr Esfahani, Dong Van Nguyen, Lam-Son Phan Tran

**Affiliations:** ^1^Signaling Pathway Research Unit, RIKEN Center for Sustainable Resource ScienceYokohama, Japan; ^2^National Key Laboratory for Plant Cell Technology, Agricultural Genetics Institute, Vietnam Academy of Agricultural SciencesHanoi, Vietnam; ^3^Department of Biology, Lorestan UniversityKhorramabad, Iran

**Keywords:** chickpea, NAC transcription factors, differential expression, differential drought tolerability, RT-qPCR

## Abstract

Drought causes detrimental effect to growth and productivity of many plants, including crops. NAC transcription factors have been reported to play important role in drought tolerance. In this study, we assessed the expression profiles of 19 dehydration-responsive *CaNAC* genes in roots and leaves of two contrasting drought-responsive chickpea varieties treated with water (control) and dehydration to examine the correlation between the differential expression levels of the *CaNAC* genes and the differential drought tolerability of these two cultivars. Results of real-time quantitative PCR indicated a positive relationship between the number of dehydration-inducible and -repressible *CaNAC* genes and drought tolerability. The higher drought-tolerant capacity of ILC482 cultivar vs. Hashem cultivar might be, at least partly, attributed to the higher number of dehydration-inducible and lower number of dehydration-repressible *CaNAC* genes identified in both root and leaf tissues of ILC482 than in those of Hashem. In addition, our comparative expression analysis of the selected *CaNAC* genes in roots and leaves of ILC482 and Hashem cultivars revealed different dehydration-responsive expression patterns, indicating that *CaNAC* gene expression is tissue- and genotype-specific. Furthermore, the analysis suggested that the enhanced drought tolerance of ILC482 vs. Hashem might be associated with five genes, namely *CaNAC02, 04, 05, 16,* and *24*. *CaNAC16* could be a potential candidate gene, contributing to the better drought tolerance of ILC482 vs. Hashem as a positive regulator. Conversely, *CaNAC02* could be a potential negative regulator, contributing to the differential drought tolerability of these two cultivars. Thus, our results have also provided a solid foundation for selection of promising tissue-specific and/or dehydration-responsive *CaNAC* candidates for detailed *in planta* functional analyses, leading to development of transgenic chickpea varieties with improved productivity under drought.

## Introduction

Drought has been considered as a major environmental constraint commonly encountered by plants, which cause significant losses to crop yield (Shao et al., 2009; Stolf-Moreira et al., 2011; Osakabe et al., 2013). Intensive research conducted in the past two decades has provided an insight into molecular mechanisms that control plant responses to drought (Shao et al., 2008; Ni et al., 2009; Hadiarto and Tran, 2011; Jogaiah et al., 2013; Albacete et al., 2014; Shanker et al., 2014). Various transcription factors (TFs) and their DNA binding sites, the so-called *cis-*acting elements, have been identified as molecular switches of stress-responsive gene expression (Yamaguchi-Shinozaki and Shinozaki, 2006; Tran et al., 2007). Among the TF families, the plant-specific NAC [no apical meristem (NAM), *Arabidopsis* transcription activation factor (ATAF), and cup-shaped cotyledon (CUC)] TF family members have been intensively studied owing to their functions in a wide range of biological processes in plants, including regulation of plant responses to environmental stimuli (Olsen et al., 2005; Tran et al., 2010; Nakashima et al., 2012; Puranik et al., 2012). Increasing number of reports have shown convincing evidence correlating drought tolerance of various plant species and expression of *NAC* genes (Tran et al., 2004; Hu et al., 2006; Nakashima et al., 2007; Thao et al., 2013; Thu et al., 2014a), suggesting their potential for genetic engineering of improved drought-tolerant crop varieties.

Chickpea (*Cicer arietinum* L.) is a nutritionally important legume crop cultivated in many countries in the Asian–African region, supplying a great source of mineral-, vitamin-, protein-, and carbohydrate-rich food for animal feeding and human consumption (Rubio, 2005; Bampidis and Christodoulou, 2011; Jukantil et al., 2012; Ngwe et al., 2012). However, drought imposes a detrimental impact on chickpea productivity worldwide, leading to a significant yield loss which has necessitated the load of chickpea research programs with the aim to develop drought-tolerant chickpea cultivars (Molina et al., 2008; Jain and Chattopadhyay, 2010; Nasr Esfahani et al., 2014). Seeing the great potential of the NAC TFs in conferring plant tolerance to drought, we recently took the advantage of the availability of the chickpea whole genomic sequence (Jain et al., 2013; Varshney et al., 2013) to identify all the *CaNAC* genes annotated in the chickpea genome (Ha et al., 2014). A total of 71 and 62 potential *CaNAC* genes was identified in the genome of the sequenced chickpea “kabuli” and “desi” cultivars, respectively (Jain et al., 2013; Varshney et al., 2013), many of which showed dehydration-responsive patterns, suggesting their involvement in regulation of drought responses in chickpea, and thus potentially playing important roles in chickpea adaptation to drought stress (Ha et al., 2014).

In this study, we further examined the functions of *CaNAC* genes in chickpea by comparing the expression levels of a subset of *CaNAC* genes in two chickpea cultivars with contrasting drought tolerance using real-time quantitative PCR (RT-qPCR) under normal and dehydration conditions. Such correlation analysis of expression levels, dehydration-responsive expression patterns and drought-tolerant degrees will enable us to identify *CaNAC* genes that are potentially associated with drought tolerance for in-depth *in planta* functional characterization prior to using them in genetic engineering for development of transgenic chickpea, as well as other crop, cultivars with superior yield under water-limited conditions.

## Materials and Methods

### Plant Growth, Treatments, and Collection of Tissues

Seeds of chickpea (*Cicer arietinum* L.) drought-sensitive Hashem and drought-tolerant ILC482 “kabuli” cultivars were received from International Center for Agricultural Research in the Dry Area (ICARDA), Syria. Hashem was developed by the Seed and Plant Improvement Institute, Karaj, Iran (Sabaghpour et al., 2005), whereas ILC482 was released by ICARDA, Syria (Singh et al., 1992). The drought-tolerant ILC482 and drought-sensitive Hashem cultivars used in this study are well-known for their contrasting drought tolerance. Their differential drought tolerability was demonstrated by the comparison of the stress tolerance index (STI), geometric mean productivity (GMP), mean productivity (MP), and harmonic mean (HM) that were determined based on their yields obtained from a field study under irrigated (well-watered) and rainfed (drought stress) conditions (Rozrokh et al., 2012, 2013). For treatments, 9-days-old chickpea seedlings grown in pots containing vermiculite under greenhouse conditions (continuous 30°C temperature, photoperiod of 12 h/12 h, 150 μmol m^-2^ s^-1^ photon flux density and 60% relative humidity) as described by Ha et al. (2014) were used. The plants were carefully removed from pots, gently washed to remove soil from roots, then subjected to either dehydration or water (control) treatments for a period of 2 and 5 h according to the methods published earlier (Tran et al., 2009). For dehydration treatment, washed plants were dried on Kim Towels (Nippon Paper Crecia Ltd.) papers, while for water treatment plants were kept in water for indicated time points. Subsequently, leaf and root samples of three biological replicates were carefully collected and frozen in liquid nitrogen for expression analysis.

### RNA Isolation, DNaseI Treatment, cDNA Synthesis

Total RNA was purified from collected leaf and root samples using RNeasy Plant Mini Kit and QIAcube system (Qiagen) according to the manufacture’s instruction. Determination of RNA concentration, DNaseI digestion, and cDNA preparation for real-time quantitative PCR (RT-qPCR) were performed as previously described (Le et al., 2011a).

### RT-qPCR and Statistical Analyses

Gene-specific primers, which were designed by Ha et al. (2014; **Table 1**), were used in the RT-qPCR analysis of 3 biological replicates to assess the expression of 19 selected dehydration-responsive *CaNAC* genes under various treatment conditions. Detailed information about the RT-qPCR reactions was described in (Le et al., 2011a). The RT-qPCR reactions were run using Stratagene MX3000P system (Agilent Technologies, Santa Clara, CA, USA) with the following thermal profile: 95°C for 1 min, 40 cycles at 95°C for 15 s and at 60°C for 1 min. After the last PCR cycle, the melting curves were obtained using the thermal profile of 95°C for 1 min followed by a constant increase in the temperature between 55 and 95°C. The *IF4a* gene, with specific RT-qPCR primers F: 5′-TGGACCAGAACACTAGGGACATT-3′ and R: 5′-AAACACGGGAAGACCCAGAA-3′, was selected as reference gene according to a report published earlier (Garg et al., 2010), and 2^-ΔΔCt^ method was used in analysis of RT-qPCR data (Le et al., 2012). Statistical significance of the differential expression within a cultivar or between 2 cultivars under well-watered or dehydration treatment was assessed using the Student’s *t*-test (one tail, unpaired, equal variance). A gene was considered as dehydration-responsive if it had at least two-fold expression change (*P*-value < 0.05) at least at one time point under dehydration. For comparison of expression levels of *CaNAC* genes between drought-tolerant ILC482 and drought-sensitive Hashem, differential expression ratio with at least two-fold (*P*-value < 0.05) was considered as significant.

**Table 1:** Primer pairs of 19 *CaNAC* genes used in RT-qPCR analysis.

#	Gene name	Forward primers^∗^	Reverse primers^∗^
1	*CaNAC02*	CCATGGGAGCTACCAAAGAA	TTTCGATCTCTCGGGCTAAA
2	*CaNAC04*	AACAAGACCACCTGACCCTG	AATGCGTCGATTTCTCAACC
3	*CaNAC05*	CTAAGGCAACGTTCGGAGAG	TTTGGCCTAGCACCATTAGG
4	*CaNAC06*	GTCCCTTCTGTGTCCACGAT	GCTCCACCACTCTGAACCTC
5	*CaNAC16*	CACCAAAGGGCCTCAAGACAG	GCCTCATGGATCCAATTTGCCTAT
6	*CaNAC19*	AGAGGTTTGGTTTGTTGGTG	CCAAACACATGGTGAGGAAA
7	*CaNAC21*	CTTACCCTTTACCCGCTTCC	TCTTCTCCCAAATCACCTGG
8	*CaNAC24*	TGCCACCAGGTTTTAGGTTC	AATGATGGAAACAGGCAAGG
9	*CaNAC27*	GCTTTGTTTGGGGATGAAGA	ACCTGCACCAGCTGCTCTAT
10	*CaNAC40*	ACGATCCTTGGGATCTTCCT	ATATTTCCTGTCTCGTGGCG
11	*CaNAC41*	CCTGAAGAGGCAATTGACAGA	TCACCACTGCAGTCAAAGGT
12	*CaNAC43*	CACTGGTGTTCTACGCTGGA	GCCGGCTGATCTATCAACAT
13	*CaNAC44*	CCCACATGGTACTCGTACTGG	TTGCAAGCCAGAAGAAGGAT
14	*CaNAC46*	TATTGGAAGGCAACAGGGTC	TTTCTTAGGCCAACAATGCC
15	*CaNAC47*	TTTCACACGGATTCAAGCTG	ACAAATTCGTTCCACTTGGG
16	*CaNAC50*	CCCACCGATGAAGAACTTGT	TACTGGAAGGGGTGCAGAAG
17	*CaNAC52*	GCTACATCAAAGCCATGCCC	GGCCTCACTCCATTTGGGTA
18	*CaNAC57*	GTGGTATGCAGGACCAAGCA	GGTGGTGGACGATGGTGATT
19	*CaNAC67*	ACAGGAGGAGAAGCTCGGAT	TCCTCATCCCGCTTTGAACC

^∗^The primer sequences were obtained from Ha et al. (2014).

### Criteria for Selection of Potential Dehydration-Responsive *CaNAC* Genes for In-Depth *In Planta* Functional Analyses and Genetic Engineering

The method was adopted from a previously published research (Thu et al., 2014b). Briefly, the selected candidate genes could be classified into two groups based on the following selection criteria. Group 1 of candidate genes are those being considered to be potential for development of improved drought-tolerant transgenic plants using overexpression approach, if they meet one of the following criteria: (i) being dehydration-inducible in tolerant cultivar vs. unchanged in sensitive cultivar and possessing higher expression levels in the tolerant cultivar under well-watered and/or dehydration conditions, (ii) showing up-regulation tendency by dehydration in both tolerant and sensitive cultivars with higher up-regulated expression change in the drought-tolerant cultivar under well-watered and/or dehydration conditions, (iii) being up-regulated in tolerant cultivar vs. unchanged in sensitive cultivar, or up-regulated/unchanged in tolerant cultivar vs. down-regulated in sensitive cultivar. Group 2 of candidate genes are those being unchanged or down-regulated by dehydration in both cultivars and showing lower expression levels in tolerant cultivar under well-watered and/or dehydration conditions. These genes could be considered for creation of improved drought-tolerant transgenic plants using gene suppression approach, such as RNA interference (RNAi).

## Results

### Expression Patterns of Selected *CaNAC* Genes in Leaves and Roots of Drought-Tolerant ILC482 Cultivar under Dehydration

The availability of natural germplasm and genetic diversity of crop varieties provides an essential key for biotechnological programs toward abiotic stress tolerance. As a means to gain a further understanding of relevant contributions of *CaNAC* genes to drought tolerance of chickpea and to identify candidate *CaNAC* genes for transgenic study, we obtained the drought-tolerant ILC482 and drought-sensitive Hashem chickpea varieties from ICARDA for comparative expression analysis of a subset of *CaNAC* genes. In a previous study, we found that expression of 19 of 23 *CaNAC* genes examined was significantly altered in leaves and roots of the drought-sensitive Hashem chickpea plants by dehydration (Ha et al., 2014), suggesting that these genes may play an important role in drought responses of chickpea. These 19 *CaNAC* genes, representing 26.76% (19/71 *CaNAC* genes identified in chickpea genome) of the *CaNAC* members in chickpea (Ha et al., 2014), were then selected to examine whether there is a correlation between their dehydration-responsive expression patterns in the drought-tolerant ILC482 and drought-sensitive Hashem and the differential drought tolerability of these two cultivars.

**FIGURE 1:**
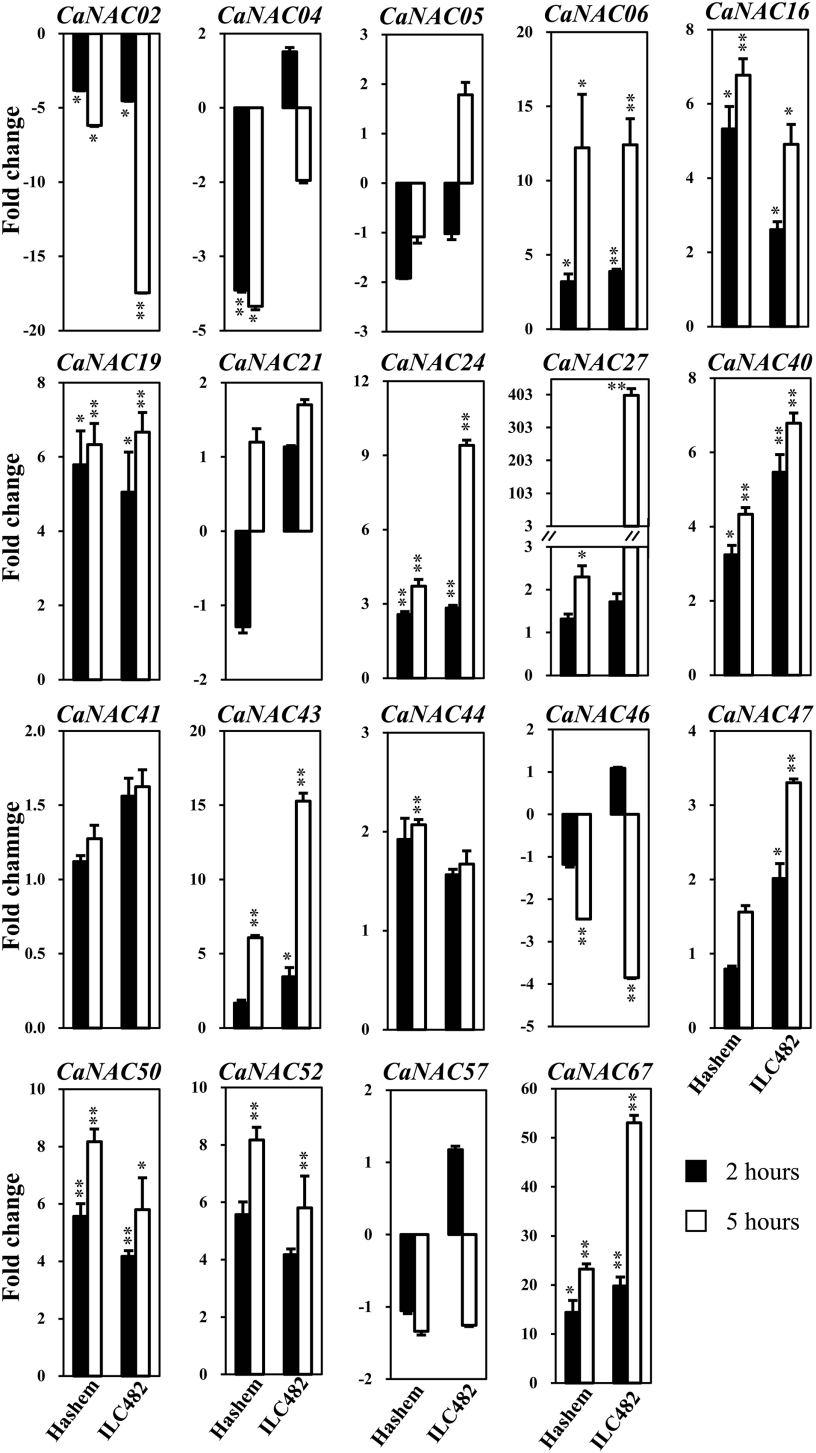
**Expression of 19 selected *CaNAC* genes in roots of drought-tolerant ILC482 and drought-sensitive Hashem cultivars under dehydration.** Expression data of the *CaNAC* genes in ILC482 roots were obtained by RT-qPCR of root samples treated with well-water control or dehydration for 2 or 5 h. For convenient comparison, expression data of the *CaNAC* genes in Hashem roots were extracted from Ha et al. (2014) and displayed. Mean relative expression levels normalized to a value of 1 in water-treated control root samples. Error bars = SE values of 3 biological replicates. Asterisks indicate significant differences as determined by a Student’s *t-*test (^∗^*P* < 0.05; ^∗∗^*P* < 0.01).

**FIGURE 2:**
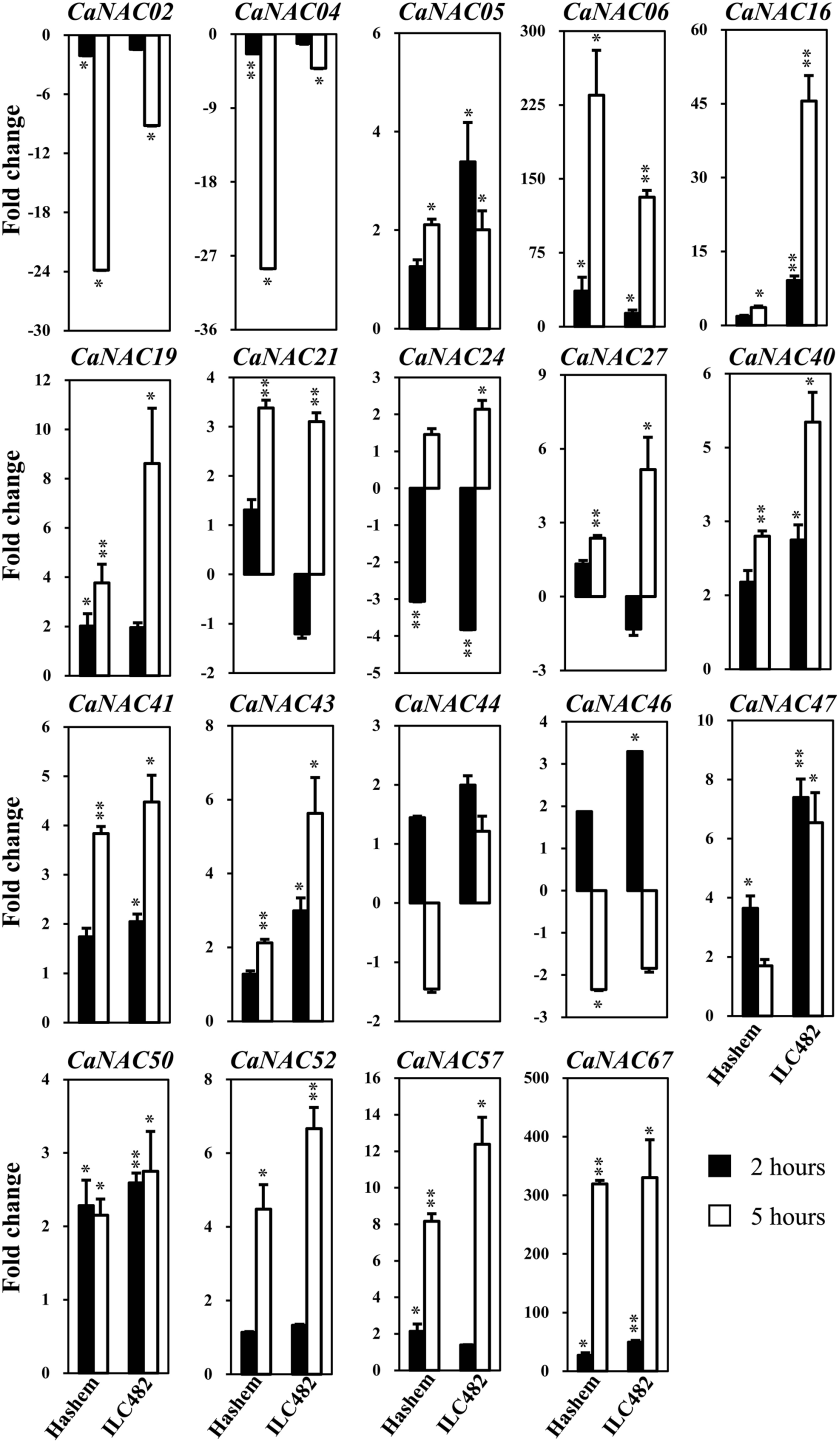
**Expression of 19 selected *CaNAC* genes in leaves of drought-tolerant ILC482 and drought-sensitive Hashem cultivars under dehydration.** Expression data of the *CaNAC* genes in ILC482 leaves were obtained by RT-qPCR of leaf samples treated with well-water control or dehydration for 2 or 5 h. For convenient comparison, expression data of the *CaNAC* genes in Hashem leaves were extracted from Ha et al. (2014) and displayed. Mean relative levels were normalized to a value of 1 in water-treated control leaf samples. Error bars = SE values of 3 biological replicates. Asterisks indicate significant differences as determined by a Student’s *t*-test (^∗^*P* < 0.05; ^∗∗^*P* < 0.01).

**FIGURE 3:**
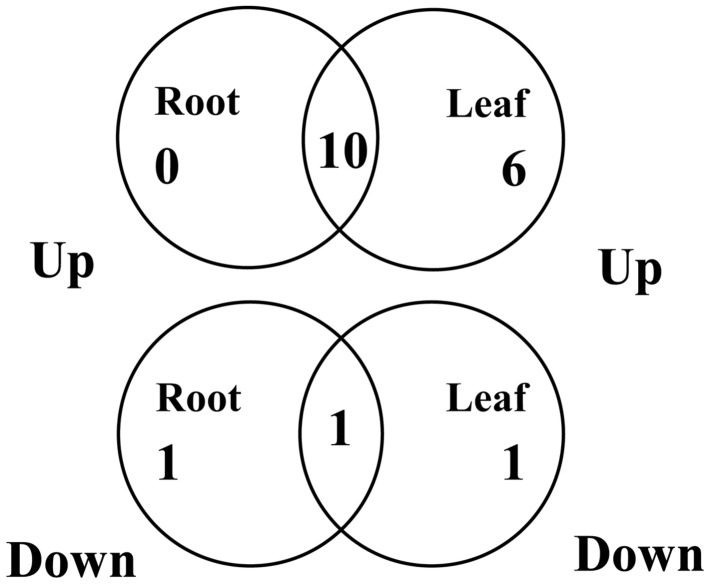
**Venn diagram analysis of expression of 19 selected *CaNAC* genes in roots and leaves of ILC482 under dehydration.** *CaNAC24* was not included in the analysis because it displayed opposite expression patterns in dehydrated ILC482 leaf tissues at 2 and 5 h.

As a first step toward this objective, we determined the expression of the 19 selected *CaNAC* genes in the leaf and root tissues of the drought-tolerant ILC482 cultivar that was grown and subjected to dehydration treatment in parallel with the drought-sensitive Hashem cultivar. All the 19 selected *CaNAC* genes also displayed dehydration-responsive in ILC482 as observed in Hashem, out of which 13 and 19 genes showed altered expression in roots and leaves of ILC482, respectively, by dehydration treatment according to the pre-defined criterion (fold-change in expression ≥ 2 and *P* < 0.05; **Figures 1** and **2**). A significant overlap was observed among the dehydration-responsive *CaNAC* genes identified in ILC482 roots and leaves, with 10 and 1 genes being induced and repressed, respectively, in both root and leaf tissues (**Figure 3**).

Specifically, we found 11 (*CaNAC06, 16, 19, 24, 27, 40, 43, 47, 50, 52,* and *67*) and 17 (*CaNAC05, 06, 16, 19, 21, 24, 27, 40, 41, 43, 44, 46, 47, 50, 52, 57,* and *67*) up-regulated *CaNAC* genes in dehydrated roots and leaves of ILC482, respectively, whereas 2 (*CaNAC02* and *46*) and 2 (*CaNAC02* and *04*) down-regulated *CaNAC* genes in the corresponding dehydrated root (**Figure 1**; **Table 2**) and leaf tissues (**Figure 2**; **Table 3**). Noticeably, *CaNAC27* and *CaNAC67* were the two most significantly induced genes in ILC482 roots and leaves by over 300- and 400-fold, respectively, whereas *CaNAC02* was the most highly repressed gene in both roots (17.5-fold) and leaves (9.2-fold) of ILC482 after 5 h of dehydration. It is also interesting to note that *CaNAC24* displayed opposite expression patterns in dehydrated ILC482 leaf tissues at 2 and 5 h, with down-regulation of 3.8-fold at 2 h but then up-regulation of 2.1-fold at 5 h of dehydration (**Figure 2**; **Table 3**). This gene was then not included in the Venn analysis to study the overlap in expression responsiveness of dehydration-responsive genes in ILC482 roots and leaves (**Figure 3**). In addition, *CaNAC46* was noteworthy to be mentioned as its expression was repressed by 3.9-fold (at 5 h) in dehydrated ILC482 roots (**Figure 1**; **Table 2**) but induced by 3.3-fold (at 2 h) in dehydrated ILC482 leaves (**Figure 2**; **Table 3**). Such opposite dehydration-responsive expression profiles in roots and leaves indicate the diverse and tissue-specific functions of *CaNAC46* in regulation of ILC482 chickpea cultivar to drought in a way that would provide the best survival of chickpea plants under water deficit conditions.

**Table 2:** Comparison of the expression levels of 19 *CaNAC* genes in the roots of ILC482 and Hashem cultivars under normal and dehydration conditions.

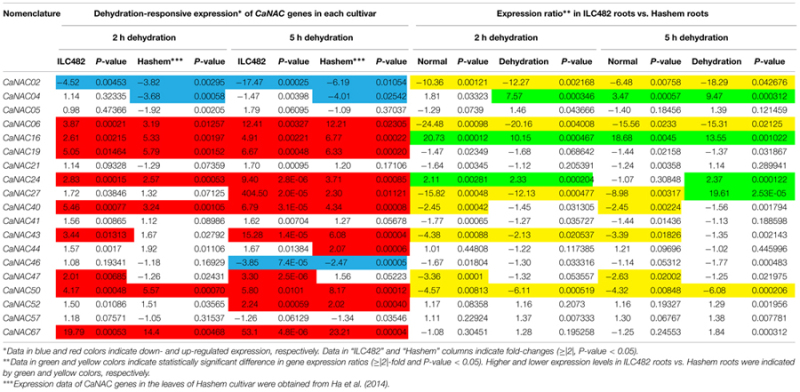

**Table 3:** Comparison of the expression levels of 19 *CaNAC* genes in the leaves of ILC482 and Hashem cultivars under normal and dehydration conditions.

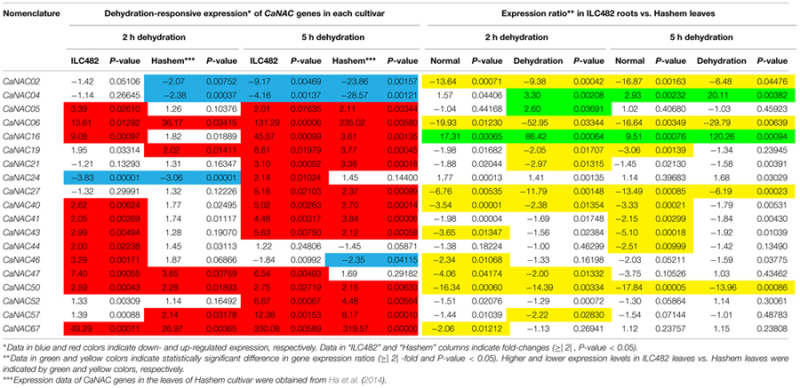

### Differential Expression of the *CaNAC* Genes in Roots of ILC482 and Hashem

As reported earlier by Ha et al. (2014), among the 19 tested *CaNAC* genes, seven (*CaNAC06, 16, 19, 24, 40, 50,* and *67*) and two (*CaNAC02* and *04*) genes were up-regulated and down-regulated, respectively, in roots of Hashem cultivar by 2 h dehydration, whereas 11 (*CaNAC06, 16, 19, 24, 27, 40, 43, 44, 50, 52,* and *67*) and 3 genes (*CaNAC02, 04,* and *46*) were induced and repressed, respectively, in the same tissues by 5 h dehydration (**Figure 1**; **Table 2**). In comparison with drought-tolerant ILC482, our data demonstrated that more *CaNAC* genes were up-regulated, whereas less *CaNAC* genes were down-regulated by dehydration in the drought-tolerant ILC482 roots than in the drought-sensitive Hashem roots. Specifically, we detected 9 and 7 dehydration-induced, as well as 1 and 2 dehydration-repressed *CaNAC* genes in roots of ILC482 and Hashem, respectively, after 2 h of dehydration (**Table 2**). As for 5 h dehydration, we recorded the same number (11) of up-regulated *CaNAC* genes in roots of ILC482 and Hashem, whereas less down-regulated *CaNAC* genes in roots of ILC482 than in roots of Hashem (2 vs. 3; **Table 2**).

A comparative analysis of expression levels of the *CaNAC* genes in the roots of drought-tolerant ILC482 vs. those in the roots of drought-sensitive Hashem revealed that under normal conditions, 2 (*CaNAC16* and *24*) and 7 (*CaNAC02, 06, 27, 40, 43, 47,* and *50*) *CaNAC* genes had higher and lower expression levels, respectively, in ILC482 roots than Hashem roots after 2 h water control treatment. The same 7 *CaNAC* genes showed lower expression levels by 5 h water treatment, while 2 *CaNAC* genes, namely *CaNAC04* and *16*, displayed higher expression levels in ILC482 roots vs. Hashem roots (**Table 2**). On the other hand, under dehydration conditions, 3 and 4 *CaNAC* genes showed higher expression levels, whereas 5 and 3 genes exhibited lower expression levels in ILC482 roots than Hashem roots after 2 and 5 h treatments, respectively (**Table 2**). Specifically, *CaNAC04, 16*, and *24* and *CaNAC02, 06, 27, 43,* and *50* were found to possess higher and lower expression levels, respectively, in ILC482 roots than Hashem roots after 2 h water control treatment. With regard to 5 h treatment, we recorded the same three genes *CaNAC04, 16*, and *24* in addition to the *CaNAC27* showing higher expression levels, whereas *CaNAC02, 06,* and *50* displaying lower expression levels in ILC482 roots vs. Hashem roots, as in the case of 2 h dehydration treatment. With the exception of *CaNAC04*, which was down-regulated in Hashem roots by both 2 and 5 h dehydration treatments, *CaNAC16, 24,* and *27* were up-regulated by dehydration in ILC482 roots, as well as Hashem roots.

### Differential Expression of the *CaNAC* Genes in Leaves of ILC482 and Hashem

With regard to the expression of the tested *CaNAC* genes in leaves, Ha et al. (2014) reported that among 19 selected *CaNAC* genes, 6 (*CaNAC06, 19, 47, 50, 57,* and *67*) and 3 (*CaNAC02, 04,* and *24*) genes showed up-regulated and down-regulated expression, respectively, in the leaves of Hashem cultivar by 2 h dehydration (**Figure 2**; **Table 3**). On the other hand, they detected more dehydration-responsive genes in 5-h-dehydrated Hashem leaves. Namely, they found 13 (*CaNAC05, 06, 16, 19, 21, 27, 40, 41, 43, 50, 52, 57,* and *67*) and 3 genes (*CaNAC02, 04,* and *46*) displaying up-regulated and down-regulated expression patterns, respectively, in 5-h-dehydrated Hashem leaves (**Figure 2**; **Table 3**). Similar to our observation in roots, when comparing the dehydration-regulated expression patterns of the 19 tested *CaNAC* genes in the leaves of ILC482 and Hashem, we found that a higher number of *CaNAC* genes were up-regulated, whereas a lower number of *CaNAC* genes were down-regulated in ILC482 leaves than in Hashem leaves by either 2 or 5 h dehydration treatment. Specifically, we recorded 11 and 15 up-regulated *CaNAC* genes in leaves of ILC482, while only 6 and 13 up-regulated *CaNAC* genes in leaves of Hashem after 2 and 5 h dehydration treatments, respectively (**Table 3**). As for the down-regulated *CaNAC* genes, we detected 1 and 2 down-regulated genes in ILC482 leaves, whereas 3 and 3 down-regulated genes in Hashem leaves after 2 and 5 h dehydration treatments, respectively (**Table 3**).

A comparison of the expression levels of the tested *CaNAC* genes in the leaves of ILC482 and Hashem revealed similar tendency as observed in the roots. Under well-watered conditions, 9 (*CaNAC02, 06, 27, 40, 43, 46, 47, 50,* and *67*) genes showed lower expression levels, while 1 (*CaNAC16*) gene possessed higher transcript abundance in ILC482 leaves than Hashem leaves after 2 h water control treatment. The same number of genes (*CaNAC02, 06, 19, 27, 40, 41, 43, 44,* and *50*) showing lower expression levels in ILC482 leaves than in Hashem leaves by 5 h water control treatment was found, whereas 2 (*CaNAC04* and *16*) genes were recorded with higher expression levels in the same comparison. Under dehydration conditions, 9 and 4 genes were noted to have lower expression levels in ILC482 leaves than Hashem leaves after 2 and 5 h treatments, respectively. On the other hands, 3 (*CaNAC04, 05,* and *16*) and 2 (*CaNAC04* and *16*) genes showed higher transcript abundance in ILC482 leaves than Hashem leaves after 2 and 5 h treatments, respectively.

### Selection of Potential *CaNAC* Candidate Genes for In-Depth *In Planta* Characterization

As a means to propose promising *CaNAC* candidate genes for further in-depth *in planta* functional analyses, which would lead to their application in generating improved drought-tolerant transgenic chickpea plants using genetic engineering, we applied the section criteria adopted from a study published previously (Thu et al., 2014b). Among the 19 *CaNAC* genes examined in this study, 5 genes could be suggested as top priorities for functional characterizations according to the selection criteria set in the Materials and Methods. Specifically, 3 (*CaNAC04, 16,* and *24*) genes of Group 1 and 1 (*CaNAC02*) gene of Group 2 were found to be satisfied for overexpression and knock-down studies, respectively, based on the differential analysis of the root expression data. On the other hand, according to the differential analysis of the leaf expression data, 3 (*CaNAC04, 05,* and *16*) genes and 1 (*CaNAC02*) gene were noted to meet the selection criteria to be classified to Groups 1 and 2, respectively.

## Discussion

The plant-specific NAC TF family is one of the important TF families in plant kingdom, whose members play diverse functions during plant growth and development (Olsen et al., 2005; Tran et al., 2010; Nakashima et al., 2012; Puranik et al., 2012). The drought-related function of NAC genes was first discovered through the study of *ANAC019, ANAC055,* and *ANAC072* in *Arabidopsis* (Tran et al., 2004), which then has led to many other studies in different plant species, including crops. One of the best studies that reported the potential application of *NAC* genes in agriculture is the work of Hu et al. (2006), who reported that transgenic rice plants overexpressing *SNAC1* exhibited enhanced drought tolerance without yield penalty. Since then, an increasing number of studies, including transgenic or correlation analyses, have provided strong evidence for the correlation between *NAC* gene expression and drought-tolerant capacity of various crops (Nakashima et al., 2007; Zheng et al., 2009; Xue et al., 2011; Thao et al., 2013; Thu et al., 2014a; Zhu et al., 2014; Yang et al., 2015).

The root plasticity is an important root trait responding to various environmental stressors, including drought, to help plants adapt to adverse conditions. Primary root length, root biomass, and number of lateral roots are all important parameters for evaluation of drought tolerance in crops (Sharp et al., 2004; Manavalan et al., 2009; Nishiyama et al., 2011; Ha et al., 2013; Zhu et al., 2014). A recent study on *SlNAC4* gene of tomato (*Solanum lycopersicum*) has provided convincing evidence for the regulatory function of NAC TFs in modulation of root growth under abiotic stresses. Suppression of *SlNAC4* expression has resulted in hypersensitivity to drought and salt stress to *SlNAC4-RNAi* transgenic tomato plants, which was attributed to inhibition of root growth, as well as a decrease in water and chlorophyll contents (Zhu et al., 2014). Thus, studying expression of the *CaNAC* genes in roots of chickpea cultivars with contrasting drought-tolerant phenotype will enable us to determine the correlation between *CaNAC* gene expression and drought tolerability, which will subsequently aid us in identifying root trait-related *CaNAC* genes for further functional analysis. The comparative expression analysis of the 19 selected *CaNAC* genes has allowed us to detect a higher number of dehydration-inducible *CaNAC* genes (9 genes vs. 7 genes and 11 vs. 11 after 2 and 5 h dehydration treatments, respectively) and a lower number of dehydration-repressible *CaNAC* genes (1 gene vs. 2 genes and 2 genes vs. 3 genes after 2 and 5 h dehydration treatments, respectively) in the roots of drought-tolerant ILC482 than in the roots of drought-sensitive Hashem (**Figure 1**; **Table 2**). These findings suggested a correlation between drought tolerability of ILC482 and Hashem cultivars and the number of the dehydration-responsive *CaNAC* genes in their roots.

In addition, leaf-related traits, such as stomata aperture and leaf cell membrane stability, have been also well-known traits that influence drought tolerance (Kaiser, 2009; Manavalan et al., 2009; Guttikonda et al., 2014; Ha et al., 2014). Overexpression of *SNAC1* gene in rice was shown to enhance stomatal closure, thereby contributing to improved drought tolerance of transgenic plants (Hu et al., 2006). This finding suggested a close association of *NAC* gene expression and leaf-related traits. Thus, it was also our interest to examine the correlation between drought-tolerant levels of the two contrasting chickpea cultivars and expression levels of *CaNAC* genes in leaf tissues under dehydration. As shown in **Figure 2** and summarized in **Table 3**, more up-regulated *CaNAC* genes, whereas less down-regulated *CaNAC* genes were found in ILC482 leaves than in Hashem leaves. These data suggested a positive correlation between drought-tolerant degree of ILC482 and Hashem cultivars and the number of the dehydration-responsive *CaNAC* genes in leaves as well, which together with the results obtained in the roots (**Figure 1**; **Table 2**) firmly demonstrated this positive correlation. Taken together, the higher drought-tolerant capacity of ILC482 vs. Hashem might partly be attributed to their differential expression of the *CaNAC* genes in both root and leaf tissues. The more *CaNAC* genes are up-regulated and the less *CaNAC* genes down-regulated by dehydration, the higher drought-tolerant the cultivar is. In support of our results, previous studies in soybean (*Glycine max*) also identified positive correlation between the number of drought-inducible *GmNAC* genes and drought-tolerant capacity of 2 contrasting cultivars (Thao et al., 2013; Thu et al., 2014a).

From our comparative analyses of the expression of these selected 19 *CaNAC* genes, we also observed differential expression patterns between roots and leaves in the same cultivar, either ILC482 or Hashem, or between the same organs of the two contrasting chickpea cultivars (**Tables 2** and **3**). This finding suggested that the expression of *CaNAC* genes, at least of those examined in this study, is tissue- and genotype-dependent, which might then result in different phenotypes of different cultivars. Differential expression analyses of *GmNAC* genes in 3 soybean cultivars with different phenotypes also showed their tissue- and genotype-dependent expression patterns (Le et al., 2011b; Thao et al., 2013; Thu et al., 2014a,c), further supporting our observation.

One of the major aims of this study is to identify the best *CaNAC* candidate genes that have high potential for development of drought-tolerant chickpea cultivars by genetic engineering. On the basis of our analysis (**Tables 2** and **3**) and the selection criteria adopted from Thu et al. (2014b), 4 (*CaNAC04, 05, 16,* and *24*) genes belonging to Group 1, and 1 gene (*CaNAC02*) classified to Group 2 could be selected for detailed *in planta* functional analyses in model plant systems, such as *Arabidopsis*, prior to using them in genetic engineering of chickpea plants or other legume crops. *CaNAC04, 16,* and *CaNAC02* are associated with both root and leave tissues, whereas *CaNAC05* and *CaNAC24* are specifically associated with leaves and roots, respectively (**Tables 2** and **3**). All these 5 genes might potentially play important roles in conferring higher drought tolerability to ILC482 than Hashem.

Out of these 5 genes, *CaNAC16* would be the best positive regulatory candidate gene as this gene was found (i) to be induced by dehydration in both roots and leaves of both ILC482 and Hashem cultivars, and (ii) to display higher expression levels in drought-tolerant ILC482 than drought-sensitive Hashem under both normal (20.73- and 18.68-fold in roots, and 17.31 and 9.51-fold in leaves at 2 and 5 h, respectively) and dehydration (10.15- and 13.55-fold in roots, and 86.42- and 120.26-fold in leaves at 2 and 5 h, respectively) conditions (**Tables 2** and **3**). On the other hand, *CaNAC02* is a promising negative regulatory gene, as this gene was strongly down-regulated by dehydration in both roots and leaves of both 2 chickpea cultivars, and showed lower expression levels in drought-tolerant ILC482 than drought-sensitive Hashem under both normal (10.36- and 6.48-fold in roots, and 13.64- and 16.87-fold in leaves at 2 and 5 h, respectively) and dehydration (12.27- and 18.29-fold in roots, and 9.38- and 6.48-fold in leaves at 2 and 5 h, respectively) conditions (**Tables 2** and **3**). Taken together, *CaNAC16* and *CaNAC02* are highly recommended for detailed functional characterization using overexpression and knock-down approaches, respectively, with the goal to lead to their application in development of chickpea varieties with improved drought tolerance.

## Author Contributions

L-SPT conceived research and wrote the manuscript. KHN, CVH, YW, UTT, and MNE performed the experiments. DVN contributed research materials.

## Conflict of Interest Statement

The authors declare that the research was conducted in the absence of any commercial or financial relationships that could be construed as a potential conflict of interest.
